# Surgical workspace in porcine thoracoscopy with two-lung ventilation

**DOI:** 10.1371/journal.pone.0325806

**Published:** 2025-07-31

**Authors:** Willem van Weteringen, Jan-Wiebe H. Korstanje, Lonneke M. Staals, Patricia A.C. Specht, Egbert G. Mik, René M.H. Wijnen, John Vlot

**Affiliations:** 1 Department of Pediatric Surgery, Erasmus MC Sophia Children’s Hospital, University Medical Center Rotterdam, Rotterdam, The Netherlands; 2 Department of Anesthesiology, Erasmus MC, University Medical Center Rotterdam, Rotterdam, The Netherlands; 3 Department of Pediatric Anesthesiology, Erasmus MC Sophia Children’s Hospital, University Medical Center Rotterdam, Rotterdam, The Netherlands; 4 Laboratory of Experimental Anesthesiology, Department of Anesthesiology, Erasmus MC, University Medical Center Rotterdam, Rotterdam, The Netherlands; University Hospital of Modena (Italy), Respiratory Diseases Unit, ITALY

## Abstract

In neonatal and pediatric thoracoscopy, two-lung ventilation is often used due to the size constraints of double-lumen tubes or selective bronchial blockers. Reducing the volume of both lungs to create surgical workspace requires moderation in the application of capnothorax insufflation pressures, and requires experienced anesthesiologists to manage ventilation. This balance was investigated in anesthetized pigs in the left decubitus position with a capnothorax, using volume-guaranteed intermittent positive pressure ventilation. End-expiratory computed tomography scans were obtained in 10 pigs (median weight 21.5 kg, range 17.8 to 26.3 kg) during incremental CO_2_ insufflation pressures of 0, 3, 5, 6, 8 and 10 mmHg. Capnothorax, right lung and left lung volumes were measured. At an insufflation pressure of 10 mmHg, peak ventilation pressures had a median of 35 cmH_2_O. Insufflation pressures ≥ 6 mmHg had profound cardiorespiratory effects, requiring inotropic support. Capnothorax volume reached a median of 1503 (IQR 1465–1596) ml at 10 mmHg, at which diaphragmatic displacement contributed 79.5% to capnothorax volume, with smaller contributions from lung volume (16.1%) and thoracic expansion (4.4%). Thoracoscopic workspace during two-lung ventilation originates mainly from diaphragmatic displacement. In a porcine model the marked cardiorespiratory consequences of insufflation emphasized the need to minimize insufflation pressures.

## Introduction

In the past decades minimal access surgery (MAS) has become the standard of care in almost all fields of surgery and age groups [1–4]. The creation of a surgical workspace inevitably involves the insufflation of pressurized carbon dioxide (CO_2_) gas into the body cavity of interest [5–8]. While in most patients the effects are well-tolerated, in certain patient groups and procedures marked cardiorespiratory side-effects occur [9–16]. Understanding the pathophysiological mechanisms involved [17] and optimizing surgical conditions is essential in these cases. While thoracoscopic procedures can generally be performed without significant problems in older children and adults, they can be challenging in patients with respiratory impairment and young children [18] in terms of surgical [19–21] and anesthesiological [22–25] management.

During thoracoscopy either a single lung or both lungs can be ventilated [26–31]. Single-lung ventilation is commonly preferred, as it allows for the collapse of the non-ventilated lung to increase surgical exposure. Despite the widespread use, there are certain limitations and drawbacks to single-lung ventilation. The required use of double-lumen tubes or bronchial blockers is associated with a risk of airway damage with potentially severe consequences [32]. These risks are even greater with the delicate airways of children. For younger children no double-lumen tubes or bronchial blockers are available [33,34]. Capnothorax with two-lung ventilation is most often the only minimal access method available for these patients.

Our research aimed to quantify thoracoscopic workspace during two-lung ventilation as a function of capnothorax pressure and determine its main constituent factors. The capnothorax volume change can originate from several contributing factors, such as lung volume change or thoracic expansion. The only applicable method for obtaining the required accurate volumetric data and anatomical measurements is computed tomography (CT) scanning [35,36]. As this is not feasible in a clinical setting, this was studied in an experimental animal model [37,38].

## Materials and methods

### Animals

A juvenile Landrace pig model was chosen, with a targeted weight of 20 kg. To minimize heterogeneity only female pigs of approximately 10 weeks of age were included, all from a single supplier. A total of 10 animals was considered sufficient to investigate the primary outcome of this study. The animals were acclimatized for 5–7 days in an enriched environment with food until the morning of the experiment and free access to water. Animals were excluded when showing any signs of illness or aberrant behavior prior to the experiment.

### Ethics statement

Animal handling and study procedures were performed in accordance with the Dutch Animal Testing Act and standard operating procedures. The institutional animal ethics committee of Erasmus MC, University Medical Center Rotterdam, approved the study protocol (EMC 3299).

### Anesthesia

The pigs were premedicated with an intramuscular injection of midazolam (1 mg/kg) and ketamine (30 mg/kg). After 15 minutes the auricular vein was cannulated, through which anesthesia was maintained with continuous intravenous perfusion of sufentanil (4 mcg/kg/h) and propofol (12 mg/kg/h). Upon confirmation of adequate anesthesia with nociceptive stimuli and the absence of the corneal reflex, a tracheotomy was performed. Tracheotomy was preferred over tracheal intubation to minimize the risk of stomach distention due to oral intubation or esophageal malplacement [39], as stomach distension influences workspace measurements. After successful tracheotomy with a cuffed single-lumen endotracheal tube, mechanical ventilation was started with intermittent positive pressure ventilation (IPPV) (fabian HFO, ACUTRONIC Medical Systems AG, Hirzel, Switzerland). The expiratory tidal volume (V_te_) was set to 10 ml/kg and volume guarantee mode, with a positive end-expiratory pressure (PEEP) of 5 cmH_2_O and a starting ventilation frequency of 35 breaths per minute. The tidal volumes were selected to guarantee adequate oxygenation and ventilation when challenged with high insufflation pressures. A fixed PEEP of 5 cmH_2_O was chosen to make the insufflation pressure the primary volume-determining factor on CT analysis. End-tidal CO_2_ values were maintained between 4.5 and 6.0 kPa (CAPNOSTAT 5, Philips Respironics, Pennsylvania, United States) by adjusting the ventilation frequency. The fraction of inspired oxygen (FiO_2_) was adjusted to maintain peripheral oxygen saturation (SpO_2_) values above 96%. After ventilation was started, an arterial cannula was inserted into the femoral artery under ultrasound guidance using the modified Seldinger technique. This was followed by a central venous catheter in the femoral vein. The body temperature was kept within the physiological range (38.0–40.0 °C) by placing the pig on electric heating pads. Ventilator parameters, core temperature, blood pressure, pulse oximetry values and electrocardiography (ECG) were all registered continuously throughout the experiments.

### Study protocol

The pig was placed in the left lateral decubitus position and a 5 mm inner diameter trocar (VersaPort™ V2 bladeless trocar, Covidien, Dublin, Ireland) was placed in the 7th or 8th intercostal space. After endoscopic confirmation of correct placement of the trocar, the pig was transferred to the CT scanner (SOMATOM Force, Siemens Healthcare GmbH, Erlangen, Germany). A CO_2_ insufflator (Endoflator 264305 20, KARL STORZ GmbH & Co. KG, Tüttlingen, Germany) was attached to the trocar. A baseline CT scan with a 1 mm slice thickness was made at 0 mmHg insufflation pressure during end-expiratory breath-hold. The thorax was then insufflated in steps (3, 5, 6, 8 and 10 mmHg), with intervals of 10 minutes to ensure a steady state in blood gas levels and end-expiratory lung volume. At each pressure step a CT scan was made, again during end-expiratory breath-hold. Inotropic support by continuous infusion of adrenaline was provided when needed to maintain adequate circulation with a systolic blood pressure of ≥ 80 mmHg and a diastolic blood pressure of ≥ 40 mmHg. When hemodynamic stability was compromised, no further pressure increments followed. After completion of the protocol the animal was euthanized by intravenous administration of a 10 ml potassium chloride 15% bolus while still under general anesthesia.

### Outcome parameters

#### Vital parameters.

The cardiopulmonary impact of thoracic insufflation was registered with ventilation parameters (V_te_/kg, PEEP, peak inspiratory pressure (PIP), FiO_2_, etCO_2_), vital parameters (heart rate, blood pressure, SpO_2_) and arterial blood gas sampling (PaO_2_, PaCO_2_). Data are presented as median with the interquartile range (IQR) unless otherwise indicated.

#### Linear dimensions.

Analysis included both linear workspace dimensions (measured with Horos™ software, Horos Project) and volumes (measured with Myrian®, Intrasense™, Montpellier, France). To determine the degree of asymmetry due to one-sided insufflation, mediastinal shift was determined by measuring the position of the carina relative to the lateral wall of the pleural cavity. Additionally, the distance between the pleural cupula and pleural sinus was measured on both sides.

#### CT volume segmentation.

The three-dimensional volumes of the intrathoracic insufflation gas volume, total lung volume and total intrathoracic volume were segmented and quantified for each animal and each insufflation pressure. The intrathoracic CO_2_ gas volume was segmented automatically using a predefined Hounsfield Unit range. Total lung volume was quantified manually by drawing each separate 1 mm slice, as atelectasis caused by thoracic insufflation did not allow reliable automatic segmentation at most pressures. Dense structures such as blood vessels up to the lung hilum were included in the total lung volume, discernible bronchi were excluded. The total intrathoracic volume was measured by adding the intrathoracic CO_2_ gas volume to the total lung volume. The total volume of the heart and large blood vessels was considered to be constant in these analyses.

#### Sources of capnothorax volume.

The main potential contributors to capnothorax volume were a reduction of the total lung volume, mediastinal shift, thoracic expansion, and diaphragmatic displacement. Aligning the scans at different pressures from each separate animal and overlaying the three-dimensional volumes allows subtraction of these volumes, distinguishing the volumetric contribution of each component to the creation of thoracoscopic workspace. For each animal the thoracic volume at a certain insufflation pressure was aligned with and overlaid onto both the previous step and the baseline measurement at 0 mmHg, and subtracted. This subtraction of volumes identifies the changes caused by an increase in insufflation pressure. Radially, thoracic volume expansion can be determined, while caudally the volume change due to diaphragmatic displacement can be distinguished.

## Results

### Animals and anesthesia

**Fig 1:**
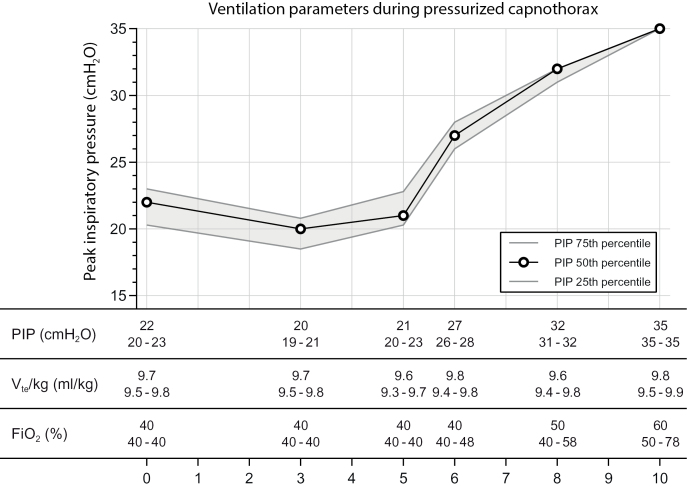
Ventilation parameters during IPPV under influence of intra-abdominal pressure. Peak inspiratory pressure (PIP, cmH_2_O), measured expiratory tidal volume per kg (V_te_/kg, ml/kg) using a volume guarantee of 10 ml/kg and the measured inspired fraction of oxygen (FiO_2_, %). All values are presented as median (IQR).

Experiments were performed in a series of 10 juvenile Landrace pigs with a median weight of 21.5 kg (range 17.8 to 26.3 kg). At insufflation pressures of 6 mmHg and higher inotropic support was needed to maintain an adequate blood pressure in all animals. Systolic blood pressures were kept constant throughout the insufflation steps, until an insufflation pressure of 10 mmHg was reached: systolic median of 75 (68.8 to 78.5) mmHg and diastolic median of 39.0 (35.5 to 41.5) mmHg. Ventilation remained stable, while oxygenation required an increase in FiO_2_ at an insufflation pressure of 8 and 10 mmHg (Fig 1). End-expiratory ventilation pauses during CT scanning were well-tolerated and did not result in noticeable tachycardia. No animals or data points were excluded from analyses. No adverse events such as pneumothorax, tamponade, bleeding or cardiac arrhythmia occurred. Ventilation frequencies were maintained at the pre-set frequency of 35 breaths per minute. Peak ventilation pressures markedly increased at insufflation pressures of 6 mmHg and higher, reaching a maximum of 35 cmH_2_O at an insufflation pressure of 10 mmHg (Fig 1).

### Computed tomography

**Fig 2:**
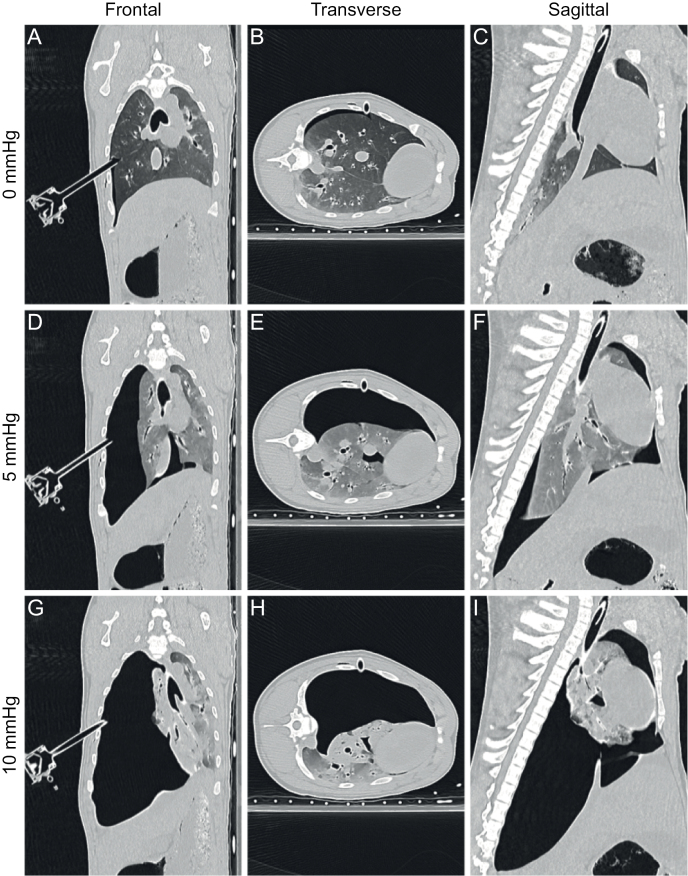
Example of capnothorax computed tomography. Computed tomography reconstructions of porcine capnothorax in a single animal with insufflation pressures of 0, 5 and 10 mmHg in the frontal (A, D, G), transverse (B, E, H) and sagittal (C, F, I) plane. The animal was placed in left lateral decubitus position. Mediastinal shift can be observed, as well as caudal diaphragmatic displacement on the side of the capnothorax.

A total of 60 end-expiratory CT scans were generated, which showed no pre-existent anatomical abnormalities. CT scanning showed considerable lung collapse and atelectasis at insufflation pressures of ≥ 6 mmHg (Fig 2), with a corresponding gain in workspace. Caudal displacement of the diaphragm could be observed, most pronounced on the insufflated side of the thorax.

### Linear dimensions

**Fig 3:**
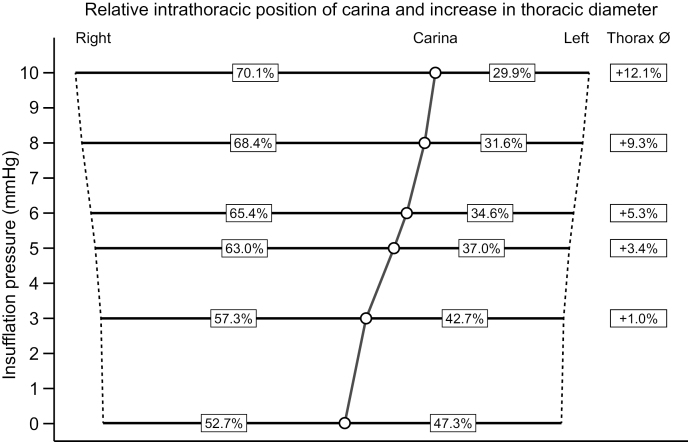
Relative changes in linear dimensions. The relative change in distance (%) between the right and left thoracic wall and the carina. The total relative increase in the thoracic transverse diameter is shown on the right. The effects of capnothorax were most evident on the insufflated side of the thorax.

Expansion of the thoracic cage due to insufflation is shown in Fig 3. The maximal transverse thoracic expansion at the level of the carina was 12.1(IQR 10.4 to 13.9)% at the highest insufflation pressure of 10 mmHg. A considerable mediastinal shift can be observed. The superior vena cava diameter decreased during the insufflation series from 10.9 (IQR 10.1 to 11.9) mm at 0 mmHg to 7.5 (IQR 6.4 to 7.9) mm at a pressure of 10 mmHg. The height of the pleural cavity, quantified by measuring the cupula-sinus distance, increased markedly only on the insufflated right side of the thorax. Pleural cavity height on the right side increased by 25.8% from 264 (IQR 252–273) mm at an insufflation pressure of 0 mmHg to 332 (IQR 324–346) mm at insufflation pressure of 10 mmHg. On the left side of the thorax there was no noteworthy increase in pleural height; from 227 (IQR 209–233) mm at 0 mmHg to 239 (IQR 234–246) mm at 10 mmHg.

### Capnothorax volumes

**Fig 4:**
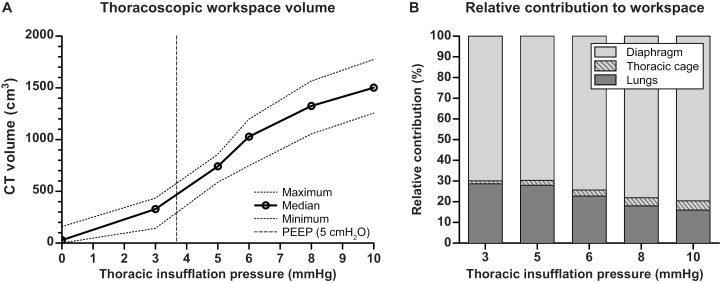
Capnothorax volumes. (A) Computed tomography capnothorax volume (cm^3^) resulting from a series of incremental insufflation pressures in 10 animals. For reference, the competing end-expiratory pressure (PEEP) is shown. (B) Relative volume contribution of CO_2_ volume, total lung volume and volume gained through diaphragmatic displacement to the resulting surgical workspace. Data are presented as the percentual contribution relative to the total lung volume at an insufflation pressure of 0 mmHg.

The relation between insufflation pressure and capnothorax volume was non-linear, with capnothorax volumes increasing markedly after surpassing the PEEP pressure (5.0 cmH_2_O = 3.7 mmHg) applied during CT scanning (Fig 4A). At an insufflation pressure of 10 mmHg, capnothorax volume reached a median of 1503 (IQR 1465–1596) ml. At that pressure the relative contributions of lung volume loss, diaphragmatic displacement and thoracic expansion to the creation of the capnothorax volume showed a large contribution of diaphragmatic displacement of 79.5%, a lesser contribution of lung volume decrease of 16.1%, and a small contribution of thoracic expansion of 4.4% (Fig 4B). Data on all individual animals can be found in supplementary S1 Table.

## Discussion

In this study we investigated thoracoscopic workspace during two-lung ventilation with capnothorax using computed tomography volume measurements in an animal model at a range of insufflation pressures. Our main conclusion was that capnothorax volume was found to originate mainly from diaphragmatic displacement in the caudal direction, to a lesser extent from lung volume reduction and to a small extent from thoracic expansion. Starting from an insufflation pressure of 6 mmHg, ventilation and at a later stage circulation were increasingly compromised in this animal model.

Capnothorax with two-lung ventilation has increased in popularity in older children and adults [30,40] as it does not have the risks associated with intubation for single-lung ventilation. Neonatal thoracoscopy poses a particular problem due to the inability to use bronchial blockers, multi-lumen tubes or other means for single-lung ventilation. It is however more difficult to create sufficient surgical workspace during two-lung ventilation. Although the application of insufflation pressures of 6 mmHg and higher can achieve more workspace, this study found the cardiorespiratory consequences to be more pronounced when compared to lower pressures, with increased ventilation pressures and a substantial decrease in blood pressure. These findings align with clinically applied insufflation pressures [41].

The insufflation of pressurized carbon dioxide gas into the thoracic cavity impairs ventilation through a shift in the balance between intrathoracic, airway, intra-abdominal and intravascular pressures. Concurrent circulatory changes also occur regularly [29]. In practice, there is a trade-off between adequate ventilation, circulation and sufficient surgical workspace [42,43]. Ventilation pressures have to overcome the insufflation pressure with each breath, intruding into the surgical workspace with each ventilation cycle. To counteract this impairment and create sufficiently large tidal volumes, relatively high insufflation pressures are commonly used. The absence of accurate clinical workspace measurements and the disregard of tissue compliance prevent a tailored approach to capnothorax. In laparoscopy there is a tipping point between the pressures at which the surgical workspace expands linearly to the applied pressure, and the pressures at which tissue is overstretched and less workspace is gained per pressure increment [38]. The thoracic cavity with its bony delineation expands markedly less with insufflation. Up to now, the competitive balance between ventilation and insufflation pressures during capnothorax has never been investigated.

Thoracic insufflation pressures are easily set to levels at which the gain in surgical workspace causes unjustifiable impairment of ventilation and circulation. Physiological heterogeneity in combination with crude insufflation pressure step settings allow only limited tailoring of insufflation pressures to the individual patient. The inability to measure surgical workspace peroperatively has therefore led to standardized insufflation and ventilation pressures, which are consequentially not optimal. A guideline on the need for using insufflation pressures that are as low as possible, as exists for laparoscopy, should be considered for thoracoscopy [44]. There is a clear demand for measurement techniques that facilitate an individualized insufflation strategy.

This study has several noteworthy limitations. A ventilation strategy was chosen with relatively large tidal volumes, which allowed for adequate ventilation and oxygenation even at high insufflation pressures. This did not affect volumetric CT analysis, performed during an end-expiratory breath hold, but should not be considered a safe strategy in clinical care, especially in patients that are ventilated for a longer duration. In addition, barotrauma can be reduced with the use of pressure-controlled ventilation with volume guarantee, which was not available for this study. The applied PEEP during insufflation will allow for lung collapse and subsequent shear stress when the difference with the insufflation pressure is too large. In this study the PEEP was kept constant to permit quantification of the effect of insufflation pressures on intrathoracic volumes. For follow-up studies and clinical care the use of a PEEP closer to the insufflation pressure is likely to reduce lung collapse at expiration and sheer stress.

The use of an animal model always has specific limitations, in the case of this porcine model the thorax-abdomen ratio differs slightly from that in humans, as well as the pulmonary anatomy, with an extra lobe on the right side. Due to the absence of evidence supporting the use of neuromuscular blockade this was not applied in these studies. It can be argued that this lack of evidence also does not exclude potential benefits, so the use of neuromuscular blockade should be considered for the investigation of surgical workspace. Another limitation of this study is the absence of more advanced cardiopulmonary monitoring such as esophageal pressure measurements, pulse pressure variation or pulse contour analysis, which might be beneficial for understanding the impact of thoracic insufflation.

Clinically important is the specific application of the results of this study in two-lung ventilation. With single-lung ventilation, facilitated by selective bronchial blockade or a double-lumen endotracheal tube, the collapse of the contralateral lung creates an entirely different pressure competition between ventilation and insufflation that deserves separate investigation.

The interesting outcome of this study is that most workspace is gained from caudal displacement of the diaphragm. This can only be properly translated to its clinical implications by understanding the physiological balance between intrathoracic, intrapulmonary and intra-abdominal pressures. Shifting this balance by increasing the intrathoracic pressure to a level higher than the physiological intra-abdominal pressure leads to a gain in thoracoscopic workspace through the path of least resistance. In this study muscle relaxation was not applied, potentially causing a specific distribution of the effects of thoracic insufflation by maintaining a diaphragmatic muscle tension. Despite this approach, the main gain in thoracoscopic workspace was obtained from the abdominal compartment. Intrapulmonary pressures are typically higher than other competing pressures, allowing adequate deployment and thus ventilation as long as peak pressures surpass the applied intrathoracic insufflation pressure. A major clinical concern that rises from this study is the use of low positive end-expiratory pressures, which are quite often set to approximately 5 cmH_2_O. Although an end-expiratory lung volume can be maintained with these intrapulmonary pressure levels in normal situations, the introduction of a competing insufflation pressure leads to expiratory lung collapse at insufflation levels higher than the intrapulmonary pressure. The same arguments that have made the open lung concept and the use of a positive end-expiratory pressure mainstay in ventilation management hold true here, with the purpose of maintaining lung aeration in the presence of a competing pressure and preventing atelectasis and alveolar sheer stress.

Counterintuitively, this study suggests that an increase in positive end-expiratory pressure can lead to improved ventilation during thoracoscopy. This practice is applied by some anesthesiologists, although this implementation of the open lung concept in thoracoscopic surgery has to date not been evaluated in clinical studies. Narrowing the bandwidth between expiratory and inspiratory pressures however decreases tidal volumes, which can impede anesthesiological management in certain patient groups. Decreased lung movement however also has beneficial effects on the stability of surgical workspace view. The use of high-frequency oscillatory or jet ventilation might therefore provide a more suitable ventilation method with the presented results in mind, decreasing lung movement even further and preventing lung collapse by maintaining a mean airway pressures higher than the applied insufflation pressures [45,46]. Identification of diaphragmatic displacement as the main constituent of surgical workspace in thoracoscopy with two-lung ventilation is suggestive of the possibility of improving conditions by changing the intra-abdominal pressure. The value of improvements in clinical care over the past decades, such as the open lung concept for ventilation or the introduction of minimal access surgery, has been proven both clinically and scientifically. The interactions between ventilation and insufflation should however be investigated, improving both thoracoscopic and laparoscopic procedures in patients of all ages and sizes.

## Conclusions

Capnothorax volumes for thoracoscopy are created mainly by caudal displacement of the diaphragm, and to a lesser extent by lung compression. The competitive balance of pressures between insufflation, ventilation and circulation resulted in difficulties in maintaining normal ventilation and blood pressures at insufflation pressures of 6 mmHg and higher. These effects emphasize the need to use the lowest possible insufflation pressure during thoracoscopy.

## Supporting information

S1 Table Measurements on capnothorax and total lung volumes. Data on all individual animals included in this study, with measured weight on the day of the experiment, and intrathoracic van total lung volumes measured on computed tomography scans made at insufflation pressures of 0, 3, 5, 6, 8 and 10 mmHg.
(DOCX)
