# Supplementary material for: Surgical workspace in porcine thoracoscopy with two-lung ventilation
Source: PLoS One. 2025 Jul 31;20(7):e0325806. doi: 10.1371/journal.pone.0325806 (PMC12312924; doi:10.1371/journal.pone.0325806)
Supplement: S1 Table — Data on all individual animals included in this study, with measured weight on the day of the experiment, and intrathoracic van total lung volumes measured on computed tomography scans made at insufflation pressures of 0, 3, 5, 6, 8 and 10 mmHg. (DOCX) [file pone.0325806.s001.docx]

**S1 Table. Measurements on capnothorax and total lung volumes.** Data on all individual animals included in this study, with measured weight on the day of the experiment, and intrathoracic van total lung volumes measured on computed tomography scans made at insufflation pressures of 0, 3, 5, 6, 8 and 10 mmHg.

| **Animal** | **Weight** | **0 mmHg** | | **3 mmHg** | | **5 mmHg** | | **6 mmHg** | | **8 mmHg** | | **10 mmHg** | |
| --- | --- | --- | --- | --- | --- | --- | --- | --- | --- | --- | --- | --- | --- |
|  | **kg** | **ITV** | **TLV** | **ITV** | **TLV** | **ITV** | **TLV** | **ITV** | **TLV** | **ITV** | **TLV** | **ITV** | **TLV** |
| 1 | 22.3 | 159 | 591 | 403 | 488 | 813 | 375 | 1050 | 346 | 1361 | 339 | 1580 | 329 |
| 2 | 22.4 | 15 | 598 | 283 | 491 | 700 | 388 | 980 | 350 | 1296 | 344 | 1511 | 337 |
| 3 | 17.9 | 21 | 506 | 348 | 425 | 757 | 321 | 944 | 314 | 1090 | 310 | 1255 | 296 |
| 4 | 21.2 | 99 | 576 | 426 | 462 | 858 | 378 | 1132 | 338 | 1410 | 331 | 1601 | 314 |
| 5 | 26.3 | 22 | 616 | 374 | 501 | 789 | 394 | 1171 | 376 | 1566 | 358 | 1774 | 352 |
| 6 | 20.0 | 0 | 522 | 215 | 429 | 606 | 313 | 747 | 304 | 1054 | 298 | 1305 | 294 |
| 7 | 22.4 | 80 | 576 | 310 | 456 | 679 | 344 | 1009 | 330 | 1316 | 327 | 1495 | 324 |
| 8 | 21.7 | 35 | 559 | 290 | 451 | 728 | 330 | 922 | 322 | 1248 | 316 | 1456 | 317 |
| 9 | 20.7 | 91 | 588 | 435 | 466 | 839 | 348 | 1197 | 349 | 1455 | 341 | 1629 | 337 |
| 10 | 17.8 | 0 | 492 | 142 | 414 | 588 | 327 | 1053 | 316 | 1335 | 299 | 1491 | 281 |

*ITV* Intrathoracic volume (ml), *TLV* Total lung volume (ml).
